# Draft Genome Sequence of *Romboutsia maritimum* sp. nov. Strain CCRI-22766^T^, Isolated from Coastal Estuarine Mud

**DOI:** 10.1128/genomeA.01044-17

**Published:** 2017-10-12

**Authors:** Andrée F. Maheux, Dominique K. Boudreau, Ève Bérubé, Maurice Boissinot, Frédéric Raymond, Stéphanie Brodeur, Jacques Corbeil, Gale Brightwell, Dorota Broda, Rabeea F. Omar, Michel G. Bergeron

**Affiliations:** a Centre de Recherche en Infectiologie, Axe Maladies Infectieuses et Immunitaires, and Centre de Recherche du CHU de Québec, Université Laval, Québec City, Québec, Canada; b Centre de Recherche en Données Massives, Université Laval, Québec City, Québec, Canada; c Département de Microbiologie, Infectiologie et d'Immunologie, Faculté de Médecine, Université Laval, Québec City, Québec, Canada; d Département de Médecine Moléculaire, Université Laval, Québec City, Québec, Canada; e AgResearch Limited, Hamilton, New Zealand; f Half Moon Bay, Auckland, New Zealand

## Abstract

The *Romboutsia maritimum* sp. nov. CCRI-22766^T^ strain was isolated from coastal estuarine mud in New Zealand. The genome assembly comprised 2,854,352 bp, with 27.1% G+C content. This is the first documentation that reports the genome sequence of *R. maritimum*.

## GENOME ANNOUNCEMENT

*Romboutsia maritimum* (*Romboutsia maritimum* [ma.ri.ti’mum. N.L. masc. gen. n. *maritimum* of maritime, pertaining to the isolation source]) CCRI-22766^T^ was isolated from a coastal estuarine mud sample in New Zealand. A comparative 16S rRNA gene sequence analysis showed that the closest cultured relative of strain CCRI-22766^T^ was *Romboutsia ilealis* CRIB^T^ (97.8% identity; GenBank accession number LN555523, positions 2388619 to 2390111) (1). The genome of *Romboutsia ilealis* CRIB^T^ was also compared to that of strain CCRI-22766^T^ for genomic relatedness assessment (accession number LN555523). The genomic average nucleotide identity obtained based on BLAST (ANIb) (2) was 77.7%. Since ANIb values around 95% corresponded to the 70% DNA-DNA hybridization cutoff value for species discrimination, strain CCRI-22766^T^ represents a novel species of the genus *Romboutsia* (3).

*Romboutsia maritimum* is a rod-shaped, strictly anaerobic, and spore-forming bacterium. It grows on blood agar in 24 h at 35°C incubated under an anaerobic atmosphere, as previously described (4), forming 6-mm colonies. Genomic DNA was isolated using the BioSprint 15 DNA blood kit (Qiagen) automated with a KingFisher mL instrument (Thermo Fisher Scientific). Whole-genome sequencing of strain CCRI-22766^T^ was performed on an Illumina HiSeq 2500 instrument using SBS version 4 to sequence a 126-bp paired-end library (Nextera XT [Illumina]). A total of 22,483,311 reads were assembled *de novo* in 95 contigs using the Ray software (version 2.3.0) (5). The total genome length is 2,854,352 bp (*N*_50_, 77,093 bp), with an average G+C content of 27.1% (6). The draft genome sequence was annotated using NCBI GenBank annotation pipeline (version 4.2) and Rapid Annotations using Subsystems Technology (RAST) annotation server (version 2.0) (7). A total of 2,867 features were identified, including 36 rRNAs (complete or partial) and 56 tRNAs. Of the 2,729 putative protein-coding sequences, 1,006 were assigned as hypothetical proteins.

### Accession number(s).

This whole-genome shotgun project of *R. maritimum* CCRI-22766^T^ has been deposited at DDBJ/ENA/GenBank under the accession number NOJZ00000000. The version described in this paper is version NOJZ01000000.
